# Research progress on the mutual regulatory mechanism of circadian clock genes and autophagy

**DOI:** 10.3389/fcell.2025.1696985

**Published:** 2025-10-29

**Authors:** Yueqi Ma, Ran Yu, Xueqing Zheng, Jiahui Rao, Gaiping Shi, Yumei Ding

**Affiliations:** ^1^ Department of Stomatology, Union Hospital, Tongji Medical College, Huazhong University of Science and Technology, Wuhan, China; ^2^ School of Stomatology, Tongji Medical College, Huazhong University of Science and Technology, Wuhan, China; ^3^ Hubei Province Key Laboratory of Oral and Maxillofacial Development and Regeneration, Wuhan, China

**Keywords:** circadian rhythm, bmal1, autophagy, circadian clock genes, molecular mechanisms

## Abstract

Recent studies have highlighted the intricate relationship between the circadian rhythm, a natural biological process responsive to light and darkness, and autophagy, a mechanism crucial for maintaining cellular homeostasis. Circadian clock genes, which are pivotal in regulating our internal body clock, appear to be closely intertwined with autophagy. These genes can directly influence the expression of autophagy-related genes or modulate signalling pathways that govern autophagic processes. Conversely, autophagy also controls the expression and activity of circadian clock genes. Investigating these interactions will help elucidate how autophagy and circadian rhythms maintain cellular equilibrium and regulate physiological functions. Moreover, such studies help reveal disease mechanisms and develop potential therapeutic strategies.

## 1 Introduction

The circadian clock, present in photosensitive organisms, regulates biological rhythms synchronized with geophysical time (Panda, 2016). This clock comprises central and peripheral clocks: the central clock integrates internal and external signals, influencing the peripheral clock via hormonal and neural pathways that govern behaviour and temperature regulation in mammals (Dibner et al., 2010; Buhr et al., 2010; Brown et al., 2002). This 24-h rhythm relies on a transcriptional feedback loop involving core proteins such as BMAL1, CLOCK, PERIOD (PER1, PER2, PER3), and CRYPTOCHROME (CRY1, CRY2) (Kume et al., 1999; Gekakis et al., 1998; Shearman et al., 2000). BMAL1 binds to the CLOCK protein to form CLOCK: BMAL1 heterodimers (Partch et al., 2014), which bind to the promoters of the PER and CRY genes, promoting their transcription and forming PER: CRY heterodimers in the cytoplasm (Michael et al., 2023). PER: CRY heterodimers inhibit CLOCK: BMAL1 activity when critical concentrations are reached (Rabinovich-Nikitin et al., 2019). In addition, the nuclear receptor families REV-ERBα and REV-ERBβ, as well as the orphan nuclear receptor RORs, are involved in regulating the circadian clock (Bunney et al., 2015; Cho et al., 2012). The circadian clock orchestrates the rhythmic gene expression governing key cellular processes—including autophagy, metabolism, redox regulation, DNA repair, and cell cycle control—through molecular-level transcription-translation feedback loops (TTFLs) (Matsuo et al., 2003; Wende et al., 2016; Sancar et al., 2010). It plays a pivotal role in maintaining organismal homeostasis and in the pathogenesis of diseases, such as cancer. Importantly, the precise regulation of autophagy by the clock constitutes a critical mechanism for maintaining cellular homeostasis (Zhu et al., 2024; Patel and Kondratov, 2021).

Autophagy is a process of self-degradation and recycling in five stages: initiation, nucleation, elongation, fusion, and degradation. This process maintains the homeostasis of the cell by decomposing and removing damaged or ageing organelles and proteins in the cell (Mizushima and Komatsu, 2011). Under nutritional deficiency or other pressures, autophagy is activated and acts as a dynamic circulatory system to remove misfolded and aggregated proteins, damaged organelles, and pathogens in the cell (Kim and Lee, 2014). Nucleation is mediated by the activation of the Unc-51-like autophagy-activating kinase (ULK1) complex (Zachari and Ganley, 2017), followed by the phosphorylation of Bcl-2-interacting protein-1 (BECLIN-1) by ULK1, which acts as a scaffold. The combination of ATG14 and BECLIN-1 promotes the formation of autophagic vesicles (Fogel et al., 2013). Then, autophagosomes are induced by ATG12-ATG5, microtubule-associated protein 1A/1B-light chain 3-II (LC3-II), and p62, which are fused with lysosomes and degraded (Corkery et al., 2023). Autophagy is involved in regulating a variety of biochemical processes, including embryonic development, antigen presentation, energy metabolism, and pathogen infection clearance (Srinivasa Reddy et al., 2020). Autophagic dysregulation can lead to various diseases, including neurodegenerative diseases, cancer, heart disease, and autoimmune diseases (Wang et al., 2020a; Yang et al., 2025; Zhang et al., 2024).

The cyclic oscillation of core clock protein levels sustains the circadian rhythm. Recently, an increasing number of studies have underscored the interdependence between the circadian clock and autophagy. In addition to its role in setting the body’s internal clock, the circadian clock precisely controls the timing of autophagy by regulating the expression of key autophagy proteins and receptors. For instance, the CLOCK:BMAL1 heterodimer binds directly to E-box elements in the promoters of genes that encode essential autophagy initiators, such as ULK1 and the scaffold protein BECLIN-1. This promotes their expression during the active phase. Furthermore, circadian oscillations in the expression of the selective autophagy receptor p62/SQSTM1 facilitate the rhythmic degradation of specific protein targets, including ubiquitinated aggregates and damaged organelles. This clock-driven rhythmic 'reshaping of the subproteome’ enables cells to periodically clear potentially toxic components and recycle building blocks in anticipation of metabolic demands, thus maintaining cellular homeostasis (Mizushima and Komatsu, 2011). Conversely, autophagy can degrade both central and peripheral core clock proteins to influence the regulation of circadian rhythm. Research into the interplay between autophagy and circadian rhythms continues to evolve. Numerous reviews have extensively documented the circadian rhythm’s governance of autophagy across diverse tissues and organs, highlighting its critical involvement in energy homeostasis, glucose metabolism, and lipid metabolism (Wang et al., 2020a; Ma et al., 2012). Building on this foundation, other analyses have provided crucial insights into the underlying molecular mechanisms of rhythmic autophagy at the cellular level. These delve into specific signaling cascades, protein-protein interactions, and transcriptional regulatory networks that orchestrate this temporal control (Maiese, 2017; Kijak and Pyza, 2017; Ryzhikov et al., 2019; Kim et al., 2023). While existing reviews have effectively established the fundamental connection between the circadian clock and autophagy and documented its physiological roles, a comprehensive and mechanistically oriented analysis of their bidirectional regulation remains largely absent. This review is specifically designed to fill this critical gap by shifting the paradigm from a unidirectional to a dynamic, reciprocal perspective. We argue that a deeper understanding of this bidirectional crosstalk is essential to fully appreciate the homeostatic control it confers.

In contrast to previous works, which often prioritize the clock’s regulation of autophagy, this review places equal emphasis on the feedback actions of autophagy on the clock. We will systematically dissect the specific pathways by which core clock genes (BMAL1, PER, REV-ERB) regulate distinct phases of autophagy. Concurrently, and with novel emphasis, we will elucidate the diverse strategies (e.g., macroautophagy, chaperone-mediated autophagy) that autophagy employs to degrade clock components and modulate clock function, highlighting the dynamic consequences of this degradation for circadian oscillation. Furthermore, we will integrate these two directions by examining the pivotal role of key molecular hubs (e.g., Melatonin, HSF1, CK1α) that simultaneously interface with both systems to orchestrate their interplay—an aspect that has not been cohesively addressed elsewhere.

By providing this synthesized, bidirectional perspective, our review aims to offer a novel and more holistic understanding of the circadian-autophagy network, which we believe is crucial for unveiling new pathophysiological mechanisms and identifying potential therapeutic targets.

## 2 Overview of rhythmic autophagy

Rhythmic autophagy was initially described in 1970, marking a major convergence of two fundamental biological processes: autophagy and the circadian clock. Researchers first noted the correlation between these processes by observing fluctuations in autophagic vacuole abundance in rat livers at different times of day. This discovery suggested that autophagy occurs rhythmically, with specific times of activation governed by the circadian clock. These findings highlight the temporal orchestration of autophagy, termed rhythmic autophagy, emphasizing its regulated activation tied to circadian rhythms (Pfeifer, 1973). It is important to note that the circadian clock, and consequently rhythmic autophagy, is not solely regulated by the geophysical light-dark cycle. Other environmental and behavioral cues, known as zeitgebers, such as feeding time, can profoundly influence circadian phase, particularly in peripheral tissues. For instance, restricting food access to a specific time window can reshape the rhythmic expression of autophagy-related genes in the liver, even when the central light-dark signal remains unchanged (Vollmers et al., 2009). This demonstrates that feeding time acts as a potent zeitgeber, capable of independently resetting peripheral clocks and autophagic rhythms. Furthermore, studies have shown that insulin signaling in response to feeding can drive the synthesis of the core clock protein PERIOD2 (PER2), thereby entraining circadian rhythms with feeding time (Crosby et al., 2019). This intricate crosstalk thereby significantly impacts overall metabolic health. Subsequent studies have provided evidence of circadian rhythm regulation of autophagy in various peripheral organs, including the kidney, liver, brain, and retina (Rabinovich-Nikitin et al., 2019; Kijak and Pyza, 2017; Ryzhikov et al., 2019; Kim et al., 2023). These extensive investigations not only reaffirmed the presence of rhythmic autophagy across diverse anatomical sites but also illuminated its broader physiological implications.

Moreover, emerging research has underscored the bidirectional relationship between autophagy and the circadian clock. Studies have shown that increased autophagic activity in the brains of mice results in a shortened circadian rhythm cycle, suggesting that autophagy plays a role in regulating circadian rhythm dynamics. This regulatory mechanism involves complex processes that include the modulation of circadian clock gene expression and the degradation of circadian clock proteins (Kim et al., 2023).

The reciprocal regulation between autophagy and the clock mechanism has been extensively investigated at the molecular level. The functional interplay between the circadian clock and autophagy is mechanistically grounded in their synchronized gene expression. This coordination is largely mediated by the core clock components BMAL1 and PER2, which, along with autophagy factors like ATG5, exhibit parallel 24-h oscillations. Crucially, BMAL1 regulates the rhythmic expression of the transcription factor C/EBPβ, which in turn acts as a master regulator by binding to the promoters of key autophagy genes such as ULK1, LC3B, and Bnip3 to drive their circadian transcription. This temporal coupling ensures that the autophagic machinery is primed and its activity is gated to the appropriate circadian phase, facilitating the timed degradation of cellular components which is crucial for tissue-specific functions and tumor metabolism (Wang et al., 2020a; Ma et al., 2012). Furthermore, clock genes activate the transcription of several crucial autophagy-related factors, including ATG14, BNIP3, and BECLIN-1, thereby regulating the cellular autophagic process (Wang et al., 2020a; Rabinovich-Nikitin et al., 2023; Lei et al., 2024). Autophagy is regulated by several clock genes, notably BMAL1, PER, and REV-ERB. Conversely, autophagy also exerts a regulatory influence on clock genes. These studies provide crucial insights into the interplay between autophagy and circadian rhythms, revealing the complex interaction between autophagy and the regulation of clock genes.

In essence, these findings underscore the intricate relationship between autophagy and circadian rhythms, highlighting the multifaceted nature of biological timekeeping mechanisms and their critical implications for organismal homeostasis and health.

## 3 Regulation of autophagy by circadian clock genes

**FIGURE 1:**
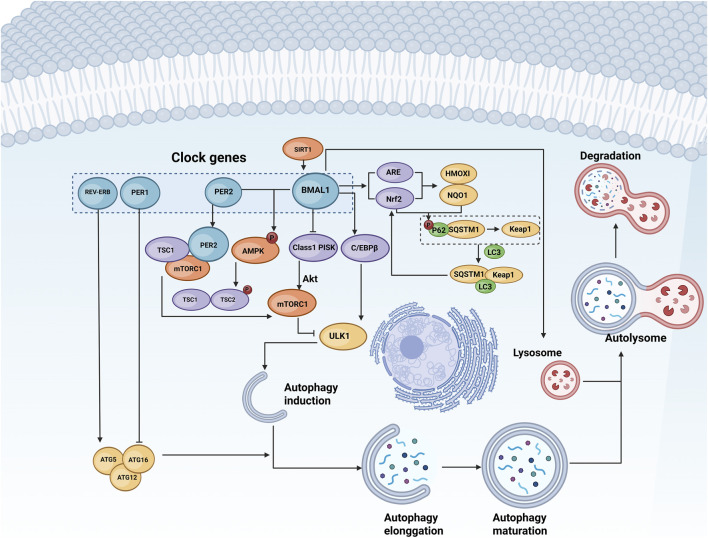
Regulation of autophagy by biological clock genes. Autophagy consists of initiation, extension, maturation, and degradation stages. Biological clock genes such as BMAL1, PERs, CRYs, and REV-ERB govern the expression and activity of autophagy-related genes such as ULK1 and ATG5 through pathways involving lysosomes, AMPK, mTOR, and other signalling molecules. This regulation influences various phases of autophagy. BioRender.

Several biological clock-related genes collaborate to maintain the stable oscillation of the molecular oscillator and the rhythmicity of autophagy, thereby ensuring the normal function of various organs in the body. Among these biological clock genes are BMAL1, PER, and REV-ERB, which play pivotal roles in regulating autophagy (Table 1; Figure 1).

**TABLE 1:** Effects of circadian clock genes on autophagy.

Circadian genes	Signalling pathway	Target	Outcome	References
BMAL1	Unknown	Lysosomal function	Upregulation	Zhao et al. (2019)
BMAL1	PI3K/Akt/mTOR	ULK1	Upregulation	McGinnis et al. (2017)
BMAL1	AMPK	TSC1/TSC, mTOR, ULK1	Upregulation	Wang and Zhang (2019), Vahsen et al. (2020)
BMAL1	C/EBPβ	ULK1, Gabarapl1, LC3B, and Bnip3	Upregulation	Xu et al. (2018), Ma and Lin (2012)
BMAL1	*Nrf*2	SQSTM1/p62	Upregulation	Bae et al. (2013)
PER1	Unknown	ATG5, ATG7	Upregulation	Kijak and Pyza (2017)
PER2	PI3K/Akt/mTOR	ULK1	Upregulation	Abe et al. (2019), Wu et al. (2019)
REV-ERB	Unknown	ULK1, Beclin1, ATG7	Downregulation	Liu et al. (2021), Huang et al. (2016)
CLOCK	Transcriptional activation	TFEB	Upregulation	Luo et al. (2016), Rabinovich-Nikitin et al. (2021)
RORα	Transcriptional activation	LC3, Beclin1	Upregulation	Kirshenbaum et al. (2023)

### 3.1 Regulation of autophagy by the BMAL1 gene

As a core component of the circadian clock, the BMAL1 gene plays a crucial role in regulating both the rhythmicity of autophagy and biological rhythms. In studies involving ischaemia‒reperfusion-induced brain injury in diabetic mice, BMAL1 was found to influence the expression of autophagy-related proteins such as P62, Beclin1, and LC3B, and this molecule also affects the formation of autophagosomes (Zhao et al., 2019). These findings further underscore the connection between BMAL1 and rhythmic autophagy. The regulatory mechanism by which BMAL1 affects autophagy can be summarized as follows.

#### 3.1.1 BMAL1 affects autophagy through lysosomal function

During autophagy, cytoplasmic material is sequestered within double-membrane autophagosomes and subsequently delivered to lysosomes for degradation. In multicellular organisms, newly formed autophagosomes undergo a process called “maturation”, wherein they fuse with vesicles derived from endolysosomal compartments, forming a double-membrane structure that eventually undergoes degradation (Yu et al., 2025). This study revealed that the accumulation of autophagosomes increased in the brains of mice with astrocyte-specific Bmal1 knockout. Additionally, pathways related to lysosomal function, independent of TFEB activation, exhibited widespread dysregulation. *In vitro* experiments further demonstrated that BMAL1-deficient astrocytes displayed increased endocytosis and lysosomal-dependent protein degradation (Zhao et al., 2021). These findings suggest that BMAL1 may regulate autophagy through its influence on lysosomal function Given that the master regulator of lysosomal biogenesis, TFEB, is itself transcriptionally activated by the core clock component CLOCK, it is plausible that BMAL1’s effects on lysosomes may be partially mediated through the circadian regulation of the autophagy-lysosome transcriptional network.

#### 3.1.2 BMAL1 regulates autophagy by regulating the mTOR signalling pathway

mTOR, a serine/threonine kinase of the PIKK family, forms two distinct complexes (mTORC1 and mTORC2) (Shimobayashi and Hall, 2014) that critically regulate diverse cellular processes, including gene transcription, metabolism (Maiese, 2014), and notably, autophagy (Szymańska et al., 2015; Dunlop and Tee, 2014; Wang and Zhang, 2019). Mechanistically, mTORC1 acts as a potent suppressor of multiple autophagic stages (nucleation, elongation, maturation, termination) primarily by phosphorylating and inhibiting the ULK1 kinase complex, a key initiator of autophagosome formation (Vahsen et al., 2020). This suppression is counteracted by AMP-activated protein kinase (AMPK). AMPK activates the TSC1/TSC2 complex, thereby inhibiting mTORC1 and relieving its repression of ULK1, thus promoting autophagy initiation (Kim et al., 2011; Shang et al., 2011).

Significantly, the activity of this mTORC1-autophagy axis is intricately linked to the core circadian clock protein BMAL1. Evidence indicates that BMAL1 exerts regulatory control over mTOR signaling: *In vivo*, mTOR activity is elevated in the brains of Bmal1 ± mice (Singla et al., 2022). *In vitro*, reduced BMAL1 expression in colorectal cancer cells correlates with increased mTOR activation (Zhang et al., 2020). Intriguingly, substantial BMAL1 overexpression in neurons also increases mTOR activity (Beker et al., 2018), coinciding paradoxically with elevated expression of mTOR negative regulators and reduced mTOR itself, suggesting complex regulatory feedback. Critically, functional linkage is demonstrated by the ability of BMAL1 overexpression to protect cardiomyocytes from hyperglycemia-induced damage; it achieves this by blocking the mTOR pathway, thereby reversing hyperglycemia-induced autophagy suppression (Qiao et al., 2017). Conversely, cardiomyocyte-specific Bmal1 knockout or clock mutation leads to overactivation of the PI3K/Akt/mTOR axis, reduced autophagy, and myocardial hypertrophy (McGinnis et al., 2017).

The circadian connection extends further to upstream mTOR regulators. AMPK itself exhibits circadian rhythmicity and is dysregulated in Bmal1(−/−) mice (Scotton et al., 2016). Furthermore, BMAL1 expression can be modulated (e.g., increased by intermittent heat stress) (Yang et al., 2023), leading to enhanced AMPK phosphorylation and subsequent autophagy induction via the AMPK/mTOR/ULK1 pathway (Yang et al., 2023). Sirtuin 1 (SIRT1), another key regulator often acting in concert with AMPK to promote autophagy (Jin et al., 2014), also interacts with this axis. SIRT1 activity negatively correlates with mTOR activity (Maiese, 2016; Maiese et al., 2013) and can inhibit mTOR under stress (e.g., oxidative stress in embryonic stem cells) (Ou et al., 2014), fostering protective autophagy. Reinforcing the BMAL1-mTOR-autophagy nexus, studies in diabetic rats show that SIRT1 activation increases BMAL1 expression and inhibits mTOR, mitigating myocardial reperfusion injury (Tang et al., 2024; Qiu et al., 2022).

In summary, BMAL1 serves as a critical nexus integrating circadian timing with the mTOR signaling pathway, predominantly through mTORC1, to exert precise temporal control over the cellular autophagic process, with AMPK and SIRT1 acting as key modulators within this regulatory network. The physiological relevance of this BMAL1-mTOR connection is particularly highlighted in extreme environments such as prolonged spaceflight. The spaceflight environment, characterized by factors like microgravity and altered light-dark cycles, is known to disrupt circadian rhythms. Crucially, a ground-based study simulating the long-duration spaceflight environment (which included circadian rhythm disruption) demonstrated a significant inhibition of the PI3K-Akt-mTOR pathway in the brain, which was associated with cognitive impairment (Wu et al., 2017), This provides direct evidence that circadian alterations in spaceflight can impact mTOR activity, underscoring the importance of this axis in maintaining physiological homeostasis under challenging conditions.

#### 3.1.3 BMAL1 regulates autophagy by regulating Nrf2 antioxidant pathway

The core circadian transcription factor BMAL1 directly regulates the Nrf2 antioxidant pathway. BMAL1 binds to E-box elements within the Nrf2 promoter and the promoters of genes containing Antioxidant Response Elements (AREs) (Pekovic-Vaughan et al., 2014). This binding drives the rhythmic transcriptional activation of Nrf2 and its downstream cytoprotective target genes, such as heme oxygenase-1 (HMOX1) and NAD(P)H quinone dehydrogenase 1 (NQO1) (Tonelli et al., 2018). Consequently, BMAL1 deficiency disrupts Nrf2 rhythmicity and impairs cellular antioxidant defenses, increasing susceptibility to oxidative stress.

Activated Nrf2 promotes the expression of Sequestosome 1 (SQSTM1/p62). SQSTM1/p62 acts as a critical bridging factor connecting Nrf2 signalling to autophagy. Under oxidative stress, SQSTM1/p62 is phosphorylated, enhancing its affinity for Kelch-like ECH-associated protein 1 (Keap1), the primary repressor of Nrf2. Phosphorylated SQSTM1/p62 competitively binds Keap1, displacing Nrf2 and facilitating Nrf2 stabilisation and nuclear translocation. Simultaneously, SQSTM1/p62 recruits microtubule-associated protein 1A/1B-light chain 3 (LC3) to form an LC3-SQSTM1-Keap1 ternary complex (Shimobayashi and Hall, 2014; Maiese, 2014; Szymańska et al., 2015). This complex is subsequently targeted for degradation via selective autophagy (specifically, SQSTM1/p62-mediated autophagy), thereby further reducing Keap1 levels and amplifying Nrf2 activation (Lin et al., 2013). This creates a positive feedback loop where Nrf2 activation promotes SQSTM1/p62 expression, and SQSTM1/p62 enhances Nrf2 activity through Keap1 sequestration and autophagic degradation (Ning et al., 2023).

Thus, BMAL1’s circadian control of Nrf2 initiates a cascade involving SQSTM1/p62 that integrates the antioxidant response with autophagy, crucial for maintaining redox homeostasis and degrading damaged components.

#### 3.1.4 BMAL1 regulates autophagy by regulating C/EBPβ

CCAAT/enhancer-binding protein β (C/EBPβ), a transcription factor, plays a pivotal role in regulating apoptosis and autophagy (Xu et al., 2018) and is crucial for activating autophagy during rhythmic oscillations. C/EBPβ is emerging as a critical factor in the regulation of rhythmic autophagy-related gene expression, with its own expression fluctuating in accordance with circadian rhythm oscillations (Ma and Lin, 2012). Additionally, C/EBPβ regulates autophagy by binding to the promoters of autophagy-related genes, thereby inducing their transcription and promoting autophagosome‒lysosome fusion (Barakat et al., 2016). Direct evidence of C/EBPβ′s role in regulating the circadian rhythm of autophagy comes from *in vivo* RNAi knockout studies. After adenovirus-mediated RNAi knockout of C/EBPβ, the circadian regulation of autophagy is disrupted. Furthermore, in the livers of BMAL1-deficient mice, the amplitude of C/EBPβ mRNA oscillation is significantly reduced. This alteration affects the expression and protein degradation of autophagy-related genes such as ULK1, Gabarapl1, LC3B, and Bnip3 (Ma et al., 2011). These studies have shown that BMAL1 can orchestrate the rhythmic regulation of autophagy by influencing C/EBPβ. However, the precise mechanism by which BMAL1 regulates C/EBPβ remains unclear.

### 3.2 Regulation of autophagy by the PER gene

The PERIOD family of repressor proteins, comprising PER1, PER2, and PER3, are encoded by their respective genes (Per1, Per2, Per3). These proteins share a common function. Among these, PER2 often exhibits the most robust oscillations and plays a particularly prominent role in regulating autophagy. These proteins can form heterodimers with cryptochrome (CRY) proteins, inhibiting and blocking the transcriptional activity of the CLOCK:BMAL1 complex (Badiu, 2003). Among these genes, PER2 shows the most pronounced oscillations and cellular localization (Gul et al., 2022). Studies have indicated that the PER gene is involved in regulating autophagy, with PER2 playing an important role and PER1 influencing the rhythmic expression of autophagy-related genes.

In wild-type flies, ATG5 and ATG7 are rhythmically expressed in the brain. However, in flies with a mutated period (per) gene, this rhythmicity is abolished Furthermore, research in *Drosophila* has been pivotal in elucidating the clock-autophagy nexus. A key study by Kijak and Pyza demonstrated that the TOR signaling pathway and autophagy are integral to regulating circadian rhythms in the fly brain. They showed that inhibiting autophagy disrupts circadian behavioral rhythms and the structural plasticity of specific neurons, establishing a direct functional link between these two fundamental processes (Kijak and Pyza, 2017).In mouse fibroblasts, siRNA-mediated downregulation of PER2 results in a substantial decrease in LC3 protein expression and a reduction in autophagy levels. This study identified PER2 as a regulator of autophagy (Kalfalah et al., 2016). In the context of folate deficiency, deprivation of folic acid can markedly increase PER2 expression through a pathway mediated by glucocorticoid receptors. This upregulation subsequently increased the expression of related autophagy genes and augmented autophagic activity (Sun et al., 2016). One week after the mice were fed during either the inactive phase (daytime feeding, DF) or the active phase (nighttime feeding, NF), the temporal expression of the Per2 gene in the livers of the diurnal-eating mice synchronized with the feeding cycle but not in the skeletal muscle. Furthermore, the Per2 gene influences the expression of the autophagy-related genes LC3B and Bnip3 (Abe et al., 2019). The mTOR pathway plays a crucial role in how the PER gene regulates autophagy. PER2, acting as a scaffold protein, interacts with tuberous sclerosis complex 1 (TSC1), Raptor, and mTOR, specifically by inhibiting the activity of the mTORC1 complex. The absence of PER2 results in the loss of mTORC1 inhibition and a decrease in autophagic activity. During fasting, in the mouse liver, glucagon-CREB/CRTC2 signalling cascades induce PER2 expression, further inhibiting mTORC1 activity (Wu et al., 2019). The role of the PER gene in tumours also involves the regulation of autophagy. The overexpression of Per2 inhibits the progression of oral squamous cell carcinoma by activating autophagy through the inhibition of the PI3K/AKT/mTOR pathway (Liu et al., 2020). In studies exploring the relationship between the PER2 gene and ovarian cancer development, PER2 was found to modulate autophagy by interfering with the PI3K/PKB signalling pathway. PER2 overexpression reduces autophagy levels, decelerates ovarian cancer growth, and inhibits tumour angiogenesis (Wang et al., 2016). Imbalances in autophagy levels resulting from disrupted oscillations and the expression of clock genes such as PER1 and PER2 are implicated in the pathogenesis of chronic obstructive pulmonary disease (COPD). Hence, the relationship between the circadian rhythm and autophagy may lead to a novel strategy for managing and treating COPD (Hu et al., 2021).

### 3.3 Regulation of autophagy by the REV-ERB gene

REV-ERBs are a family of nuclear receptors that include REV-ERBα and REV-ERBβ. These receptors can bind to retinoic acid-related orphan receptor elements (ROREs) in the BMAL1 promoter, thereby inhibiting its transcription (Bugge et al., 2012). *In vitro* studies have demonstrated that REV-ERB agonists can suppress autophagy and induce apoptosis. *In vivo* experiments have shown that REV-ERB agonists downregulate the mRNA and protein expression of autophagy-related genes such as Ulk3, Ulk1, Beclin1, and Atg7, leading to significant inhibition of glioblastoma growth *in vivo* (Sulli et al., 2018). Both REV-ERBα and REV-ERBβ regulate autophagy. In studies investigating the functional impact of REV-ERBα on hepatic steatosis, the inhibition of REV-ERBα expression in the mouse liver promoted autophagy. Similarly, *in vitro* experiments with increased REV-ERBα expression in L-02 human normal liver cells revealed that increased REV-ERBα expression inhibited cellular autophagy activity, exacerbating steatosis. These findings suggest that REV-ERBα promotes the progression of alcoholic fatty liver (AFL) by suppressing autophagy and inducing hepatic lipid degeneration (Liu et al., 2021). Luciferase reporter gene and ChIP experiments have demonstrated that the circadian clock can directly control the expression of autophagy-related genes in zebrafish larvae through REV-ERBα (Huang et al., 2016). Studies have also revealed that REV-ERBα inhibits the transcription of ATG5 by binding to the RORE cis-element in its promoter, thereby suppressing autophagy (Zhang et al., 2022). The regulation of autophagy by REV-ERBβ occurs downstream of the autophagy blockade pathway. This process also serves as a determinant of sensitivity to the clinically relevant anti-autophagy lysosomal drug chloroquine (De Mei et al., 2015).

### 3.4 Regulation of autophagy by the CLOCK gene

CLOCK, as a core transcription factor of the circadian clock, plays a pivotal role in regulating autophagy, particularly mitochondrial autophagy (mitophagy). It forms a heterodimer with BMAL1 to activate the transcription of downstream targets. A key mechanism involves CLOCK’s direct activation of the TFEB promoter. As a master regulator of lysosomal biogenesis and autophagy, TFEB coordinates the expression of genes essential for autophagosome-lysosome fusion and substrate degradation, thereby governing the final stage of the autophagic pathway. Notably, TFEB itself is under circadian control, exhibiting rhythmic expression in mouse livers, and its interaction with the CLOCK/BMAL1 complex is potentiated by glucose levels, positioning TFEB as a key molecular nexus through which nutrient and circadian signals converge to regulate autophagic flux (Luo et al., 2016).

During hypoxic stress, overexpression of CLOCK has been shown to restore mitophagy and mitochondrial biogenesis in cardiac myocytes, as evidenced by increased lysosomal clearance of mitochondria and elevated levels of the biogenesis regulator PPARGC1A. This demonstrates that CLOCK is integral to the circadian coordination of mitochondrial quality control via autophagy (Rabinovich-Nikitin et al., 2021).

### 3.5 Regulation of autophagy by the RORα gene

Retinoic acid-related orphan receptor alpha (RORα) is another circadian component that positively regulates protective autophagy. RORα activity declines during hypoxic stress, coinciding with impaired autophagy and increased cell death. The natural compound Nobiletin acts as a potent RORα agonist. It binds to the RORα promoter, leading to its activation and the subsequent upregulation of autophagic flux and mitophagy. This RORα-dependent activation of autophagy is crucial for clearing damaged, ROS-producing mitochondria and for promoting cell survival under stress. The cytoprotective effects of Nobiletin are abrogated upon RORα knockdown, and its function is also dependent on core autophagy machinery like ATG7, placing RORα upstream of autophagy activation in this protective pathway (Kirshenbaum et al., 2023).

## 4 Regulation of circadian clock genes by autophagy

**FIGURE 2:**
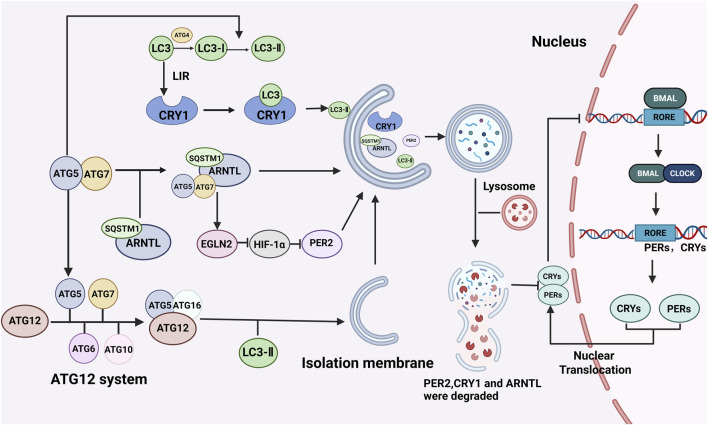
Regulation of the biological clock by autophagy. The regulatory effect of autophagy on the biological clock mainly involves the degradation of biological clock proteins. Autophagy-related proteins such as LC3 and ATG5 can bind to the corresponding receptors of the circadian clock proteins CRY1 and ARNTL, promote protein degradation, destroy the circadian clock molecular oscillator’s stable cycle, and regulate the circadian clock. BioRender.

Currently, increasing evidence has demonstrated that autophagy is regulated by circadian clock genes. However, as a crucial cellular self-degradation process, autophagy has also been shown to participate in regulating the circadian clock. The mechanisms through which autophagy influences the circadian clock mainly include the following aspects (Figure 2).

### 4.1 Autophagy regulates the expression of the core genes of the circadian clock

In U2OS cell models established via the use of autophagy activators such as rapamycin and AICAR, the mRNA levels of LC3B and ATG5 were markedly increased, whereas the mRNA levels of the clock genes BMAL1, PER2, and REV-ERB were significantly reduced. Knockout of the autophagy-related protein ATG5 attenuated the impact of autophagic activation on U2OS cell clock oscillators (Gao et al., 2023).

### 4.2 Regulatory effects of autophagy on circadian clock protein degradation

#### 4.2.1 Regulatory effects of the autophagy-related protein LC3 on circadian clock protein degradation

The periodicity of the circadian clock forms the foundation for maintaining circadian rhythms, with the degradation of circadian clock-related proteins playing a crucial role in this periodicity. As a pathway for quality control, autophagy is involved in the degradation of circadian clock proteins. Research has identified lysosomal targeting sequences in circadian clock proteins such as BMAL1, CLOCK, REV-ERBα, and CRY1 (Parico et al., 2020). As identified by Toledo et al., the CRY1 protein contains multiple putative LC3-interacting region (LIR) motifs. Subsequent site-directed mutagenesis and functional analyses revealed that, among these, two distinct LIR motifs (LIR1 and LIR4) are primarily responsible for mediating the interaction with the autophagy protein LC3 and its subsequent degradatio. This specific autophagic degradation of CRY1, an inhibitor of gluconeogenesis, plays a critical role in regulating circadian glucose metabolism. Additionally, CRY1 acts as an inhibitor of gluconeogenesis, and its degradation via autophagy increases under conditions such as a high-fat diet (Toledo et al., 2018; Zhou et al., 2024). Furthermore, the selective autophagy of CRY1 is closely linked to the regulation of blood glucose levels in the body (Tong et al., 2017). The LIR motif also contributes to the regulation of circadian blood glucose levels by influencing the degradation of CRY1, suggesting a potential target for managing hyperglycaemia. AMP-activated protein kinase (AMPK) has also been implicated in the regulation of the autophagic degradation of CRY1. In the mouse liver, the activity and nuclear localization of AMPK are rhythmically negatively correlated with the abundance of the nuclear CRY1 protein, and its phosphorylation disrupts CRY1 stability. This process promotes proteasomal degradation of CRY1, thereby altering circadian rhythms (Lamia et al., 2009; Um et al., 2007).

#### 4.2.2 Regulation of circadian clock proteins by the autophagy-related proteins ATG5 and ATG7

The autophagy-related proteins ATG5 and ATG7 play crucial roles in regulating the degradation of circadian clock proteins. Their primary function is to induce autophagosome formation and facilitate the conversion of LC3B-I to LC3B-II, a marker of autophagosome formation (Wang et al., 2020b). In RSL3-induced mouse embryonic fibroblasts (MEFs), ATG5 and ATG7 have been implicated in the degradation of ARNTL/BMAL1. The multifunctional cargo receptor SQSTM (Lamark et al., 2017), which is involved in the autophagic degradation of ubiquitinated substrates such as proteins and organelles, acts as a direct receptor for ATG5-and ATG7-mediated ARNTL degradation. After ARNTL is degraded through autophagy-associated proteins and ubiquitin-binding protein-dependent selective autophagy, the expression of human prolyl hydroxylase 2 (EGLN2) increases, and the stability of hypoxia-inducible factor-1 alpha (HIF-1α) decreases. This process may consequently lead to abnormal expression of the PER2 gene (Yang et al., 2019; Tamaru et al., 2011).

### 4.3 Regulation of the circadian clock by CMA

Chaperone-mediated autophagy (CMA) is a process in which chaperones recognize specific motifs within protein sequences, facilitating their transport to lysosomes for degradation (Kaushik and Cuervo, 2018; Dong et al., 2021; Qiao et al., 2021). Chaperone-mediated autophagy (CMA) promptly regulates the circadian clock through synergistic interactions with other proteolytic systems (Gatfield and Schibler, 2007; He, 2021). Clock proteins achieve precise levels at specific circadian times through transcriptional‒translational feedback loops and post-translational regulation. Variations in the timing and regulation of proteolytic systems dictate when and under what conditions clock proteins undergo degradation through these pathways. Loss of CMA disrupts the circadian cycle, leading to a phenotype resembling ageing. Some CMA substrates may be directed to alternative degradation pathways when CMA is impaired, but this compensatory process is insufficiently sustainable (Ryzhikov et al., 2019). Studies on the lysosomal abundance of CLOCK proteins at different time points indicate their dependence on chaperone-mediated autophagy (CMA) degradation. ARNTL/BMAL1 and CLOCK proteins predominantly undergo CMA degradation during the nighttime, whereas a substantial portion of CRY1 degradation via CMA occurs during the light period. In contrast, RORα exhibits cyclic lysosomal binding but does not undergo degradation through this pathway (Ryzhikov et al., 2019; Kaushik et al., 2022). Functionally, disruption of chaperone-mediated autophagy (CMA) in the body disrupts both central and peripheral circadian clocks. Reduced CMA activity during ageing may contribute to abnormal circadian rhythms in elderly individuals.

### 4.4 Intermittent fasting as a potent zeitgeber for circadian autophagy

Feeding-fasting cycles are potent environmental cues that synchronize circadian rhythms and autophagy. Intermittent time-restricted feeding (iTRF), which confines food intake to specific hours aligned with the active phase, robustly enhances circadian-driven autophagy. In *Drosophila*, iTRF boosts the night-time expression of core clock genes (PER, Tim) and autophagy genes (ATG1, ATG8a), leading to a timed peak of autophagic activity during the fas.Crucially, the anti-ageing benefits of iTRF, including lifespan extension and delayed tissue ageing, strictly require an intact circadian clock and functional autophagy machiner. This establishes a direct causal link. Furthermore, genetic induction of autophagy specifically during the night is sufficient to extend lifespan on an *ad libitum* diet and prevents further iTRF-mediated longevity, while day-specific induction is ineffective. This underscores that the timing of autophagy activation is critical (Ulgherait et al., 2021).

These findings have significant clinical implications. They suggest that misaligned eating can disrupt beneficial circadian autophagy, while deliberately aligned IF regimens offer a therapeutic strategy to amplify it. This approach holds potential for enhancing metabolic health by clearing damaged organelles, promoting neuroprotection through timely clearance of toxic proteins, and supporting overall healthy ageing. Thus, IF acts as a behavioural tool to harness the circadian-autophagy network for maintaining cellular homeostasis and combating age-related diseases.

## 5 Molecules that affect both the circadian clock and autophagy

**FIGURE 3:**
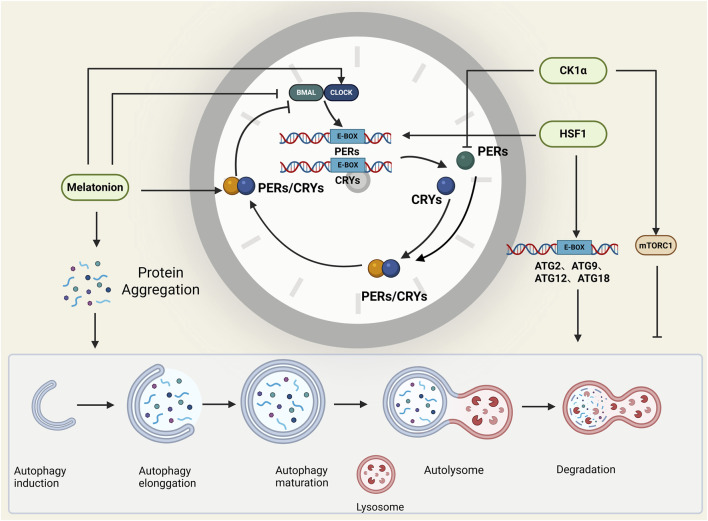
Molecules that affect both autophagy and the circadian clock. Melatonin, HSF1, and CK1α regulate both autophagy and the circadian clock, and they play important roles in the connection between the circadian clock and autophagy. BioRender.

At present, a variety of molecules have been confirmed to simultaneously regulate the circadian clock and autophagy; these molecules play important roles in the connection between autophagy and the circadian clock, and the relevant molecules mainly include the following types (Table 2; Figure 3).

**TABLE 2:** Molecules that influence both autophagy and circadian rhythms.

Molecules	Target	Outcome	References
Melatonin	PER2, CLOCK	Circadian rhythm changes	Qiao et al. (2021)
Melatonion	Elimination of misfolded protein aggregation	Promotion of autophagy	Gatfield and Schibler (2007), He, 2021; Kaushik et al. (2022), Ulgherait et al. (2021)
HSF1	PERs BMAL1:CLOCK heterodimer	Circadian rhythm changes	Reiter et al. (2016)
HSF1	SQSTM1 S349, SQSTM1 S403	Promotion of autophagy	Jung-Hynes et al. (2010)
HSF1	ATG2, ATG9, ATG11, ATG18	Promotion of autophagy	Zheng et al. (2014)
CK1α	PER1	Degradation	Shi et al. (2018)
CK1α	mTOR	Inhibition of autophagy	Hossain et al. (2021), Shen et al. (2018)

### 5.1 Melatonin

Melatonin, a hormone secreted by the pineal gland of the brain (Reiter et al., 2016), is an indole heterocyclic compound that plays an important role in the regulation of circadian rhythm and autophagy. In prostaglandin carcinoma cells (PCas), melatonin upregulates the expression of Per2 and Clock and represses the expression of Bmal1, whereas melatonin treatment results in the resynchronization of oscillatory circadian genes (Dbp and Per2) (Jung-Hynes et al., 2010). In addition, melatonin has a dual effect on autophagy; melatonin also plays a central role in the removal of misfolded protein aggregation by autophagy and can promote autophagy by inhibiting methamphetamine toxicity to prevent neuronal cell death in the brains of Alzheimer’s disease patients. In addition, the antioxidant properties of melatonin have inhibitory effects on autophagy. Therefore, in different stages of autophagy, melatonin can play a key role as an autophagy-promoting indicator or an anti-autophagy regulator (Zheng et al., 2014; Shi et al., 2018; Hossain et al., 2021; Shen et al., 2018).

### 5.2 Heat shock transcription factor 1 (HSF1)

In normal cells, various stressors, including heat, hypoxia, and toxins, activate heat shock factor 1(HSF1), resulting in increased expression of so-called heat shock or chaperone proteins to maintain stressed cell homeostasis (Mendillo et al., 2012). The receptors regulating autophagy are phosphorylated at the SQSTM1 S349 and SQSTM1 S403 residues in an HSF1-dependent manner, thus decreasing autophagy (Watanabe et al., 2017). Moreover, HSF1 can act on the promoters of the autophagy-related genes ATG2, ATG9, ATG11, and ATG18 and regulate the expression of autophagy-related proteins (Li et al., 2016). In addition, HSF1 can bind the promoter of the PER gene and interact with the BMAL1:CLOCK heterodimer to regulate the circadian clock and circadian rhythm (Tamaru et al., 2011).

### 5.3 Casein kinase 1α of the CK1 protein family (CK1α)

Casein kinase 1α (CK1α) in the CK1 protein family plays a regulatory role in several important signalling pathways involved in membrane transport, the cell cycle, chromosome segregation, apoptosis, autophagy, cell metabolism, developmental differentiation, the circadian rhythm, the immune response, neurodegeneration, and cancer (Jiang et al., 2018). CK1α has dual effects on autophagic regulation: on the one hand, the inhibition of CK1α can activate the mTOR signalling pathway to inhibit autophagy (Zhao et al., 2011; Gao et al., 2011); on the other hand, the overexpression of CK1α can increase autophagic flux in non-small cell lung cancer (NSCLC) cells (Jiang et al., 2018). Moreover, CK1α-mediated phosphorylation promotes Per1 degradation (Lam et al., 2018), the presence of which is essential for the stability of the circadian rhythm.

## 6 The existing problems

At present, some problems related to the mutual regulation of autophagy and circadian clock genes remain to be solved. First, the specific regulatory mechanisms and signalling pathways involved have not been fully defined, and further studies are needed. Second, whether there is time-dependent regulation between clock genes and autophagy and how autophagic activity at different time points affects the normal function of the clock still need to be further explored. In addition, the roles of autophagy and circadian clock genes in disease occurrence and development need to be further elucidated.

## 7 Conclusions and future perspectives

In this review, we systematically explored the molecular mechanisms underlying the mutual regulation of circadian clock genes and autophagy. Research on the regulatory mechanism between circadian clock genes and autophagy, as well as rhythmic autophagy, has important biological and medical value. Abnormal autophagic processes are closely related to the occurrence and development of metabolic diseases such as cancer, neurodegenerative diseases, and diabetes. The study of rhythmic autophagy can reveal the abnormal regulation of autophagy in cells, help alter the intracellular metabolic process, provide a new therapeutic strategy for disease treatment, and lay a foundation for the development of new therapeutic methods. A deeper understanding of rhythmic autophagy offers potential targets for drug development, and drugs that regulate autophagy may be used to treat diseases associated with abnormal autophagy. The regulation of the circadian clock by autophagy affects the physiological state of cells and organs at different points in time and is crucial for maintaining the overall physiological rhythm of the organism. Overall, the study of rhythmic autophagy contributes to a deeper understanding of the dynamic nature of intracellular regulatory networks, providing new perspectives and strategies for explaining the fundamental principles of life and treating various diseases. Research in this area is highly valuable for the discovery of new drug targets, the development of more effective treatments, and the advancement of life sciences.

Building upon this mechanistic framework, future efforts should focus on three key areas. First, employing tissue-specific circadian clock models will clarify the organ-specific functions of circadian autophagy. Second, the bidirectional regulation supports the development of chrono-therapeutic strategies, such as timing autophagy-targeting drugs to specific circadian phases to enhance efficacy in oncology and neurodegenerative diseases. Finally, time-resolved multi-omics approaches are essential to map the dynamic “circadian-autophagy regulome,” revealing novel rhythmic substrates and regulatory mechanisms for a systems-level understanding.
